# Assessing unConventional Evidence (ACE) tool: development and content of a tool to assess the strengths and limitations of ‘unconventional’ source materials

**DOI:** 10.1186/s12961-023-01080-9

**Published:** 2024-01-02

**Authors:** Simon Lewin, Etienne V. Langlois, Özge Tunçalp, Anayda Portela

**Affiliations:** 1 Department of Health Sciences Ålesund, Norwegian University of Science and Technology, Ålesund, Norway; 2 Centre for Epidemic Interventions Research (CEIR), Norwegian Institute of Public Health, Oslo, Norway; 3 Health Systems Research Unit, South African Medical Research Council, Cape Town, South Africa; 4 Partnership for Maternal, Newborn & Child Health (PMNCH), World Health Organization, Geneva, Switzerland; 5 UNDP/UNFPA/UNICEF/WHO/World Bank Special Programme of Research, Development and Research Training in Human Reproduction (HRP), Department of Sexual and Reproductive Health and Research, World Health Organization, Geneva, Switzerland; 6 Department of Maternal, Newborn, Child and Adolescent Health and Ageing, World Health Organization, Geneva, Switzerland

**Keywords:** Source materials, Grey literature, Tacit knowledge, Professional knowledge, Real world evidence, Decision-making, Methodology, Evidence synthesis, Tool

## Abstract

**Background:**

When deciding whether to implement an intervention, decision-makers typically have questions on feasibility and acceptability and on factors affecting implementation. Descriptions of programme implementation and of policies and systems are rich sources of information for these questions. However, this information is often not based on empirical data collected using explicit methods. To use the information in unconventional source materials in syntheses or other decision support products, we need methods of assessing their strengths and limitations. This paper describes the development and content of the Assessing unConventional Evidence (ACE) tool, a new tool to assess the strengths and limitations of these sources.

**Methods:**

We developed the ACE tool in four stages: first, we examined existing tools to identify potentially relevant assessment criteria. Second, we drew on these criteria and team discussions to create a first draft of the tool. Third, we obtained feedback on the draft from potential users and methodologists, and through piloting the tool in evidence syntheses. Finally, we used this feedback to iteratively refine the assessment criteria and to improve our guidance for undertaking the assessment.

**Results:**

The tool is made up of 11 criteria including the purpose and context of the source; the completeness of the information presented; and the extent to which evidence is provided to support the findings made. Users are asked to indicate whether each of the criteria have been addressed. On the basis of their judgements for each criterion, users then make an overall assessment of the limitations of the source, ranging from no or very minor concerns to serious concerns. These assessments can then facilitate appropriate use of the evidence in decision support products.

**Conclusions:**

Through focussing on unconventional source materials, the ACE tool fills an important gap in the range of tools for assessing the strengths and limitations of policy-relevant evidence and supporting evidence-informed decision-making.

## Background

Decision-makers are interested not only in whether an intervention or programme works but also how it works, for whom and under what circumstances, what the key components of the intervention are, and the critical lessons learned during implementation. When making decisions about whether and how to fund or implement an intervention or programme, decision-makers therefore typically have a wide range of questions and information needs. These include the effectiveness of the intervention; the costs associated with implementing it (and its cost-effectiveness); how feasible it is within the implementation context, such as a health or social care system; the acceptability of the intervention to key stakeholders, such as service users, health workers and managers; its likely equity and human rights impacts; and what factors might facilitate or hinder implementation. Increasingly, decision-makers are drawing on evidence from different types of systematic reviews or evidence syntheses to address these questions. Use of the best available global evidence on intervention effectiveness is now common in decision-making, including in the development of clinical and health systems guidelines at national and global levels. Decision-makers are also starting to draw on evidence from syntheses of primary qualitative studies as well as mixed methods studies, to address questions around intervention acceptability, feasibility and equity impacts and implementation considerations [1–3], as well as utilizing economic evidence more extensively [4–6].

However, given the need to better understand implementation issues and how programmes evolve and are scaled up, there is also growing interest in making greater use of documented experiences, professional or tacit knowledge [7], and ‘local evidence’ [8], including from sources such as national experiences with implementation and health management information systems (sometimes referred to as ‘real world evidence’ [9]). These descriptive, or non-empirical, types of information are often not based on empirical data collected using explicit methods and are typically not included in evidence syntheses or other summaries that may be used to inform decision-making. Real-world evidence may sometimes be part of comparative evaluations or primary qualitative studies (for instance, as part of the description of the intervention) and may also be found in programme reports in the so-called ‘grey’ or ‘professional literature’ [10] or in descriptions published on the web. Programme, implementation, policy and systems descriptions and other largely descriptive types of information are therefore potentially important sources of relevant real-world evidence.

These sources can provide documentation and insights regarding key intervention components, how an intervention works, how it might be implemented in a ‘real-world’ setting and factors affecting implementation. They can also provide information on how a policy was developed and operationalized, including the governance, financial and delivery systems arrangements used. Through providing documentation on the acceptability and feasibility of interventions and programmes, and factors that may affect implementation, these sources can also inform the development of clinical, health systems and public health guidelines at global and national levels. [11, 12]. They can also inform guideline adaptation and implementation tools at national and subnational levels [13], as in the case of the WHO antenatal care (ANC) adaptation toolkit and the upcoming WHO toolkit on postnatal care [14]. Programme, implementation, policy and systems descriptions have an advantage in relation to global guidance products: through providing contextually relevant ‘real-world’ evidence, they may enhance the credibility of these decision support products among policymakers, programme managers and clinicians [15].

Because of the recognized value of these sources to inform decision-making, efforts are underway both to improve the reporting of programme or intervention descriptions [16, 17] and to try to include this wide range of ‘non-empirical’ sources in evidence syntheses to inform decision-making. In this context, these sources can also be described as ‘unconventional’ in that they are not routinely included in evidence syntheses for decision-making.

If we are to draw on real-world evidence and these unconventional sources of information in syntheses or other products intended to inform decision-making, we need methods of appraising them to identify their strengths and limitations. This process of critical appraisal is key to assessing whether this information is reliable and trustworthy – in other words, whether the information is threatened by important risks to rigour [18]. A wide range of tools is available to critically appraise or assess the methodological limitations of different kinds of primary and secondary research (Table 1). However, there are few tools available for critical appraisal of programme descriptions, descriptions of implementation (for example, in programme evaluation reports) and other largely descriptive types of information. By ‘programme’, we mean a set of organized instructions, activities or actions intended to address or respond to a particular issue or to achieve a particular objective. For example, a workplace health programme may include a range of actions to improve the health and wellbeing of workers within a particular organization.

**Table 1:** Examples of types of empirical studies for which tools to assess methodological limitations are already available

• Randomized studies such as randomized trials and cluster randomized trials
• Quasi-experimental (non-randomized) and observational studies such as ITS, CBAs, cohort studies, case–control studies and regression analyses
• Economic evaluations
• Primary qualitative studies
• Systematic reviews of the effectiveness of interventions
• Qualitative evidence syntheses/systematic reviews of qualitative evidence

Tools exist to assess ‘grey literature’ documents, but these both include a wider and less well-defined group of sources (i.e. “document types produced on all levels of government, academics, business and industry in print and electronic formats that are protected by intellectual property rights” [19]) and are not only intended to critically appraise these sources but also to consider their possible usefulness and impacts [20, 21]. We aim to address this gap by developing a new tool to assess the (strengths and) limitations of unconventional sources of information. We believe that this tool will contribute, over time, to improving the standard of reporting of programme descriptions and other descriptive types of information that are useful for decision-making, policy and programme planning and implementation.

## Aim

To describe the development and application of a tool to guide the critical appraisal, or assessment of the methodological strengths and limitations of, source materials, including descriptions of policies and programmes and implementation descriptions.

## Methods

In this work, we have chosen to use the term ‘source material’ to refer to the types of information to which this new tool could be applied. When used in decision-making, source materials are a form of evidence – that is, “facts (actual or asserted) intended for use in support of a conclusion” ([22] p. 1). We have chosen the term ‘source material’ as some (but not all) of these materials are not empirical studies or the product of a research process but are rather generated as part of the routine planning and implementation of interventions, programmes or policies, and include some forms of data sometimes referred to as ‘real-world evidence’ [9]. Source materials may also capture expert or tacit knowledge [7, 23, 24] – ‘the observations or experience obtained from a person who is knowledgeable about or skilful in a particular area’ [25], such as service users, health workers and service managers. We also describe these sources as ‘unconventional’ – a term we have chosen to indicate that these sources are not included routinely in evidence syntheses for decision-making. While unconventional source materials encompass a wide range of different types of information, they largely share the following features:

- They are generally descriptive rather than analytic in nature.
- They are generally based on people’s views, experiences or observations and/or data from routine sources (such as a health management information system, HMIS).
- They rarely include empirical data collected using explicit methods, as would the case in a planned research study.

Table 2 provides examples of unconventional source materials. These may be available as: (i) documents such as programme reports, white papers, policy briefs, peer reviewed journal papers, etc., or as (ii) websites and other online material. It is important to note that while these types of information may be reported in ‘stand-alone’ documents, such as a report or blog, such information may also be included in papers reporting empirical studies. For instance, comparative evaluations of interventions may include detailed descriptions of how the intervention was implemented as well as reflections on factors affecting this implementation (for example, [26]). These materials may be commissioned or produced by a range of actors, including government departments, non-governmental organizations (NGOs), civil society organizations, academic institutions, multilateral and bilateral agencies and the private sector.

**Table 2:** Examples of unconventional source materials

• Descriptions of ‘real-world’ (i.e. not part of experimental studies) health, welfare or other programmes or interventions or policies or system reforms, including how they were developed
• Descriptions of the implementation of programmes or interventions or policies in the field, including pilot programmes
• Descriptions of policy processes and system reforms. This could include descriptions of how the processes or reforms were planned; contextual determinants of policy or programme implementation; and how system settings (e.g. welfare system, health system) influence the impacts of programmes, interventions or policies
• Sources that report information from routine health management and information system (HMIS) data managed by departments of health; other service delivery organizations; or sentinel sites
• Sources that report people’s views and experiences of a health or social issue, programme or policy, and that appear to be based on qualitative methods of data collection (such as interviews), but do not describe any explicit research methods and is not clear whether any formal analysis was undertaken
• Sources that report information on the basis of data from projects, including pilot projects, but without any comparative evaluation data and/or without a description of the methods used

**Fig. 1:**
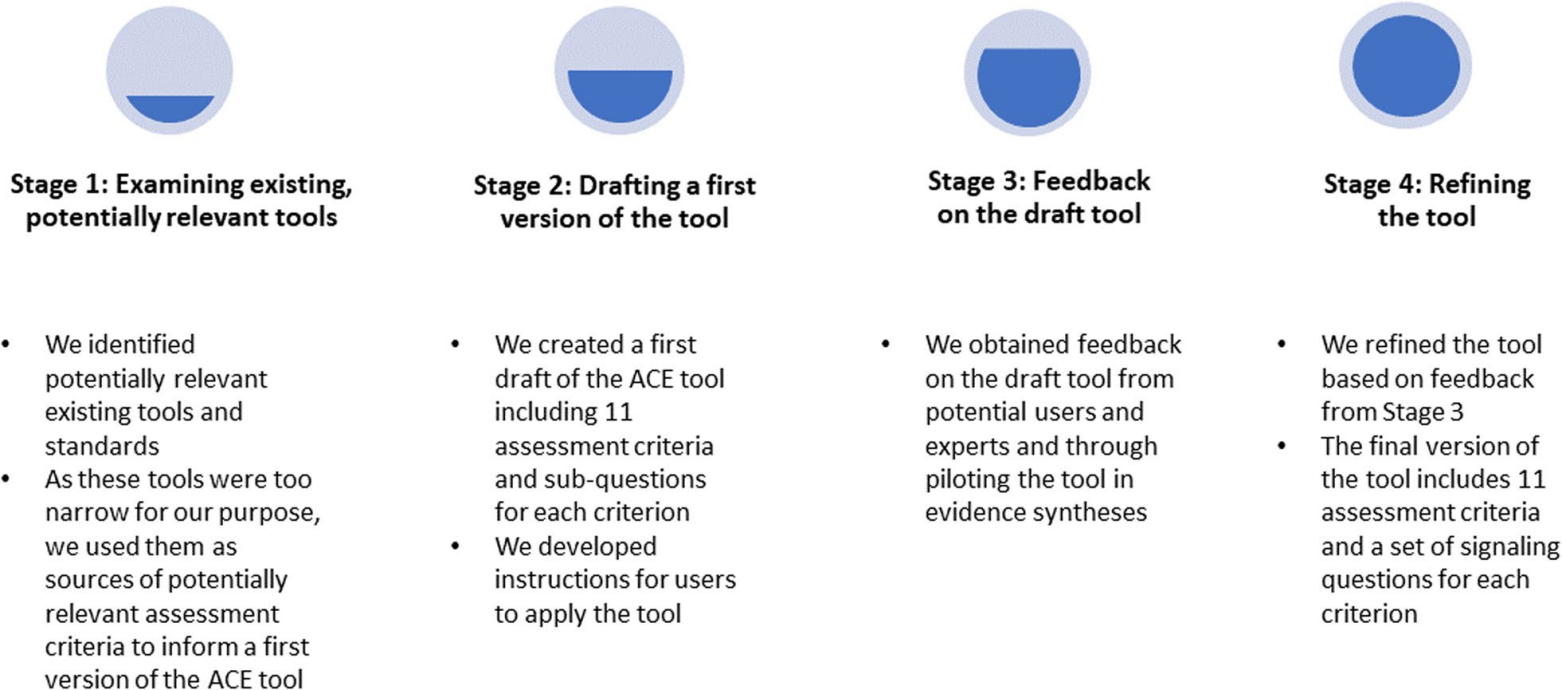
Development of the ACE tool

We developed the ACE tool in four stages (Fig. 1), as described below, using an approach similar to that used for other assessment tools that we have developed [27, 28] and drawing on the approach recommended for reporting guidelines by the EQUATOR Network [29].

### Stage 1: examining existing, potentially relevant tools

Through informal searching and contact with colleagues, we identified a number of existing tools and standards that were potentially relevant:

- The AACODS checklist for grey literature [21]
- A NICE tool for critical appraisal of grey literature [20]
- The TIDieR checklist for describing interventions [16]
- A tool developed to assess risk of bias in case studies, as part of a Cochrane review on interventions to reduce corruption in the health sector [30]
- The JBI checklist for text and opinion [24]
- The WHO Programme Reporting Standards (PRS) for sexual, reproductive, maternal, newborn, child and adolescent health (SRMNCAH) programmes [17]
- SUPPORT tool for findings and using evidence about local conditions [8]

While these tools included some useful elements/criteria, they did not fully address our needs in relation to a tool to assess the limitations of the types of source materials described above. There are several reasons for this: two of the tools focus on the so-called grey literature [20, 21]. However, there are a number of definitions of what is encompassed by the term ‘grey literature’, and these definitions generally include a wide range of empirical data and non-empirical sources that are available outside of commercial sources and/or are not peer-reviewed. For example, the Cochrane Handbook for Systematic Reviews of Interventions describes grey literature as “reports published outside of traditional commercial publishing” [31], while the Twelfth International Conference on Grey Literature defined this literature, following a review of definitions and consultations with stakeholders, as “manifold document types produced on all levels of government, academics, business and industry in print and electronic formats that are protected by intellectual property rights, of sufficient quality to be collected and preserved by library holdings or institutional repositories, but not controlled by commercial publishers…” [19]. These broad definitions of ‘grey literature’ potentially encompass empirical studies as well as many other kinds of sources. Tools for assessing the methodological limitations of many of these sources are already available, as noted above; our interest was in developing a tool specific to unconventional sources.

One of the tools, the TIDieR checklist [16], is specifically intended to improve completeness in the reporting of interventions in research studies, while another is focussed on assessing risk of bias for case studies reporting an intervention and using explicit data collection and analysis methods [30]. While useful sources of relevant assessment criteria, these tools were too narrow for our purpose. We therefore used these tools as sources of potentially relevant assessment criteria that could inform a first version of our tool for assessing the strengths and limitations of unconventional source materials.

### Stage 2: drafting a first version of the tool

Drawing from criteria described in existing tools and a series of discussions within the team, we created a first draft of the tool. This version included 11 assessment criteria and sub-questions for each criterion. Two of the criteria were intended only for source materials that include empirical data while the remaining criteria were intended for all source materials. Instructions were provided for users to select yes, no or unclear for each criterion, and then to use these judgements to make an overall assessment of the source materials using the following categories: no or few limitations; minor limitations; and significant / major limitations.

### Stage 3: feedback on the draft tool

We obtained feedback on the draft tool in two ways: firstly, we presented a draft version of the tool at a series of meetings with potential users and experts. Over a period of 18 months, we held two meetings with members of the Policymaker Network – a group convened by the Alliance for Health Policy and Systems Research that includes decision-makers from low- and middle-income countries involved in using evidence to support policy and systems decision-making and to advance universal health coverage. We also held meetings with researchers attending the Global Symposium on Health Systems Research in 2018; experienced systematic reviewers in the Division of Health Services, Norwegian Institute of Public Health; and staff involved in developing global guidelines at WHO Headquarters. Approximately 30 people in total, from a wide range of settings, backgrounds and disciplines, participated in these discussions. We used the resulting feedback from each meeting to refine the tool iteratively.

Secondly, we piloted the tool in three evidence syntheses that included unconventional sources of information [32–34]. One of the authors (SL) was a co-author on these reviews but did not lead the application of the tool. We asked the lead review author to apply the tool on the basis of the criteria and guidance developed, and to keep notes of any challenges arising from this process. SL later independently checked the assessments and discussed any disagreements with the lead authors. This feedback from the lead authors and these discussions were used to refine the tool.

### Stage 4: refining the tool

On the basis of the feedback from stage 3, we refined the tool. The main changes were as follows:

- Assessment criteria: we simplified the phrasing of the assessment criteria. For source materials that include little or no empirical data, we moved from assessing accuracy to assessing completeness of the information presented. We added a criterion on whether relevant rights and ethics considerations are described. We also refined the sub-questions and moved to calling this ‘signalling questions’ to indicate that they are intended to guide the user and that not all of the questions will be relevant to all source materials
- Assessment process: we revised the rating category options for the assessment criteria; added one additional assessment option (‘not applicable’); and suggested that users include a justification for their assessment, preferably supported by extracts from the source material/s. We also incorporated the overall assessment section into the main assessment table, expanded the range of overall assessment options, and indicated that users should provide an explanation of the overall assessment
- Guidance for undertaking the assessment: we elaborated and improved our guidance on how to make an overall assessment of the strengths and limitations of the source material/s and provided additional guidance points regarding issues such as assessing a programme described in multiple documents, the use of the signalling questions for each criterion, and how to undertake an assessment of whether relevant rights and ethics considerations were described. We also moved from using the term overall ‘limitations’ to overall ‘concerns’, to bring the tool in line with the terminology used in other tools such as the GRADE-CERQual approach [35].

## Results

### Assessment criteria included in the tool

The tool is made up of 11 criteria for assessing the limitations of unconventional source materials (Table 3). These criteria focus on the purpose and context of the source; the completeness or accuracy of the information presented; the extent to which evidence is provided to support any findings made; and aspects of reporting, such as whether relevant rights and ethics considerations are described. The tool also includes a set of signalling questions for each criterion. These are intended to help users apply the tool. Most of the criteria and signalling questions are intended to be applied to all types of sources. However, criterion 6 is intended for sources that include empirical data while criterion 5 is more relevant to sources that include little or no empirical data. A detailed description of the tool and guidance on how to apply it can be found at https://zenodo.org/doi/10.5281/zenodo.7650035.

**Table 3:** Assessment criteria included in the ACE tool

ACE assessment criteria	Summary of the signalling questions for each criterion
1. Is the aim, objective or purpose for the source material described?	Does the source material state its aim clearly or can this be derived from the material?
2. Is the source of the information reported?	Where did the information being assessed come from, and where applicable, who collected this information?
3. Is there a description of the programme or intervention or policy or reform on which the source material focuses?	What are the goals, content and target of the programme, intervention or policy; who was involved in delivering it; and how was this done?
4. Is there a description of the context/s to which the information described in the source material relates?	Does the source material describe where the programme or policy or reform took place, including aspects such as setting, health or social care system, socio-cultural context and/or political and legal context?
5. Is the information complete?	Does the source material describe any efforts to ensure that the information presented is complete and reliable?
6. Is the information accurate?	For source materials that include empirical data, are the methods for obtaining and analysing these data appropriate?
7. Is the information representative?	If the information is based on a sampling process, how was this done, was this appropriate and are any generalizations made appropriate?
8. Is information provided to support any findings or conclusions made?	Are the findings or conclusions supported by relevant information?
9. Are any limitations of the information and/or methods discussed in the source material?	Does the source material outline any gaps or weaknesses in the information provided?
10. Are relevant rights and ethics considerations described?	Does the source material discuss relevant rights and ethics considerations?
11. Are any interests declared and any potential conflicts of interest noted?	Are potential conflicts of interests described, including how these were addressed?

### Undertaking an assessment of source materials using the tool

Each tool criterion is phrased as a question, and users are asked to choose one of the following response options: yes, no, partial, unclear or not applicable. The signalling questions are intended to guide users in selecting an appropriate response option. Some of the criteria and signalling questions need to be addressed using the source as a whole (e.g. criteria 10 and 11 on rights and ethics considerations and potential conflicts of interest respectively) while other criteria and signalling questions focus on the specific information from the source material that is being used in an evidence synthesis or decision process (e.g. criterion 7: ‘Is the information representative?’). For the latter group of questions, assessment should focus on the information that is being used rather than all of the information in the source.

Users should also provide support for their judgements underlying these assessments as this improves the transparency of the process and helps readers understand the judgements made. Information to support each judgement should be drawn from the sources being assessed (see example in Table 4). On the basis of the judgements for each criterion, users should then make an overall assessment of the limitations of the source, selecting one of the following overall assessment options: no or very minor concerns; minor concerns; moderate concerns; or serious concerns. In making an overall assessment, users should look across the responses for all of the questions in the tool. If they think that the limitations identified would undermine or would probably undermine the reliability of the material, then they may want to select ‘moderate’ or ‘serious’ concerns. Where the limitations identified are small (i.e. the user has selected ‘yes’ for most criteria), and probably do not undermine the reliability of the information in the source, then they may want to select ‘minor concerns’.

**Table 4:** Example of an ACE Tool assessment (adapted from [34])

Description of the document or source	
Type of source material	Other [please describe]: press release [61]*
Pre-assessment questions	
Is the source material based on, or does it include, empirical data?	Yes

ACE assessment criteria	Assessment	Justification for the assessment
1. Is the aim, objective or purpose for the source material described?	Unclear	No stated aim, but the source intends to describe a mHealth initiative
2. Is the source of the information reported?	Partial	Some sources are described but others are not described fully
3. Is there a description of the programme or intervention or policy or reform on which the source material focusses?	Yes	“Innovative mobile application designed to make birth registration of children smart, quick and reliable in Ghana” (p. 1). “The automated birth registration system is an Android App which has been customized for Tigo Network only…[]… The tablets use the mobile app to collect data related to the child’s name, gender, date of birth and other family details, which are then sent to the central database managed by the Births and Deaths Registry. Once received, the data is stored and an automated response is sent to the Births and Deaths Registry official on the field, confirming that a certificate can be issued. Whereas data collected though the paper-based system takes six months to be registered in the central system, the mobile registration process achieves this in less than two minutes” (p. 2).
4. Is there a description of the context/s to which the information described in the source material relates?	Partial	Only some sub‐assessment criteria described, as the source notes only that the intervention was implemented in Ghana and does not provide information on the areas of Ghana that are covered by the intervention or how and by whom it was delivered
5. Is the information complete?	Unclear	The source does not describe efforts to ensure that the information is complete and accurate
6. Is the information accurate?	Unclear	The source includes some empirical data but insufficient information is provided to assess whether it is accurate (see response to Q8 below)
7. Is the evidence representative?	Unclear	The source does not provide sufficient detail to assess whether the information is complete and representative (see response to Q8 below)
8. Is information provided to support any findings or conclusions made?	Partial	The source describes implementation but includes very limited information or empirical data. The source notes that “According to analysis done by Births and Deaths Registry, UNICEF and Tigo, at the end of the one year pilot, over 670 800 new births will be registered on the new system by the end of May 2017. This would increase Ghana’s birth registration rate to 75 percent, from the previous 65 percent” (p. 2).
9. Are any limitations of the information and / or methods discussed in the source material?	No	Not described
10. Are relevant rights and ethics considerations described?	No	Not described
11. Are any interests declared and any potential conflicts of interest noted?	Partial	The organizations involved in producing the press release are described but there is no explicit declaration of interests or discussion of conflicts of interventions
Overall assessment of the limitations of the source material and explanation	Serious concerns	Concerns about limited information regarding the sources of information and the context/s described. In addition, the source material did not describe in sufficient details efforts to ensure that the information presented was complete and accurate and did not describe the limitations of the information or any relevant rights or ethics considerations

*The source material used in this example was identified and assessed as part of a systematic review of documents examining: (1) the range of strategies used to implement birth and death notification via mobile devices; and (2) factors influencing the implementation of birth and death notification via mobile devices

We recommend that two reviewers apply the tool independently to each source, and then discuss their assessments and reach a consensus assessment for each source. Where a team is involved in making ACE tool assessments, it may be valuable for team members to work together initially to assess two or three of the included source materials so that a shared approach can be developed. We do not recommend assigning numeric values or scores as the assessments are judgements and scoring is likely to give a spurious level of certainty to the assessment process.

## Discussion

**Fig. 2:**
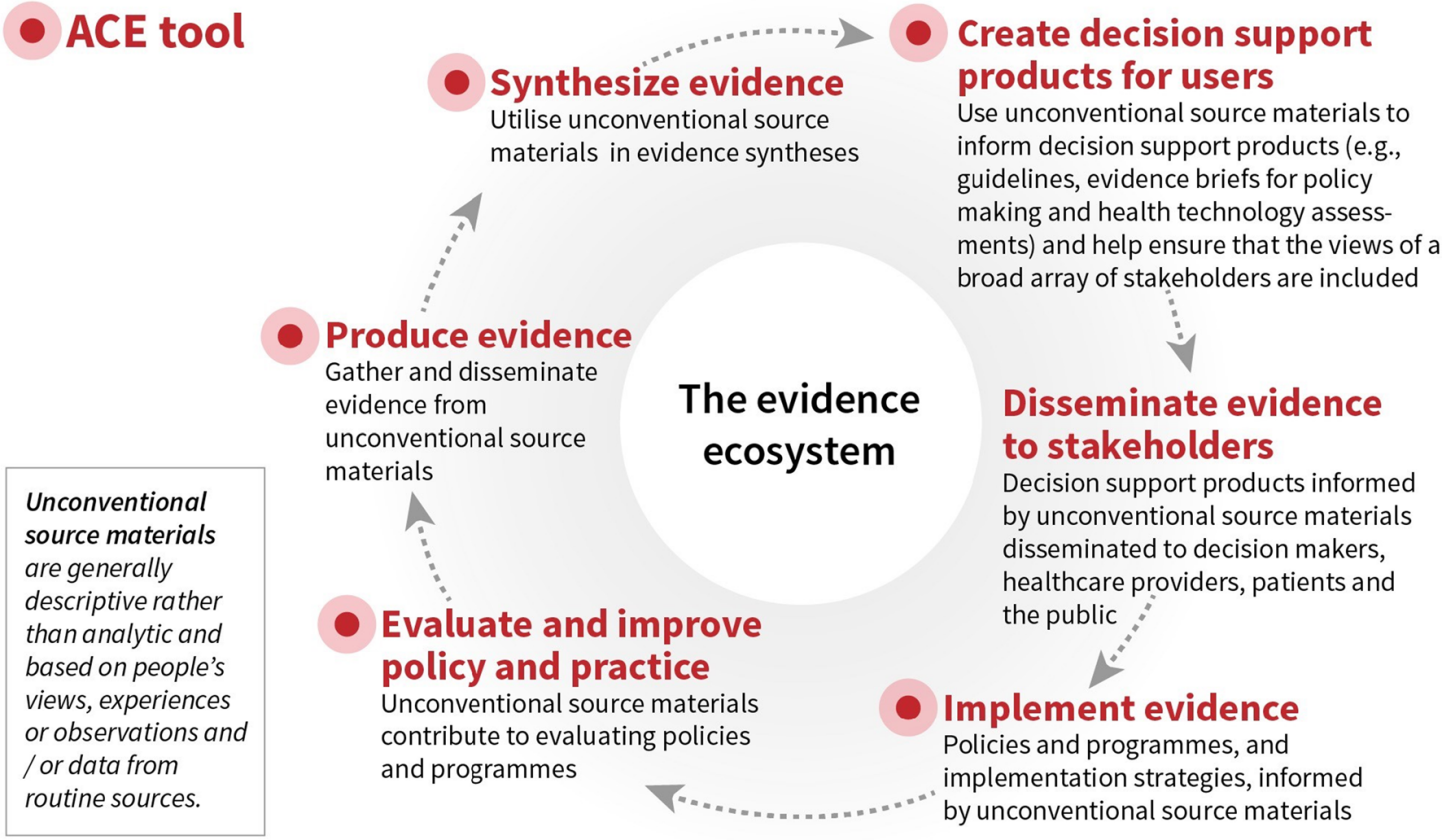
How unconventional source materials and the ACE tool can contribute to the evidence ecosystem (adapted from [62, 63])

The ACE tool provides a set of criteria to guide assessment of the strengths and limitations of the information from unconventional source materials. It allows those supporting decision-making, including the authors of evidence syntheses as well as technical teams involved in decision processes or programme implementation, to apply a systematic approach to making these assessments. The tool therefore fills an important gap in the palette of tools available to critically appraise the different types of information used to inform decisions in healthcare and other sectors. Below we discuss the use of the ACE tool in evidence syntheses, to support evidence-to-decision processes and programme implementation, and to improve the reporting of programmes. Unconventional source materials may contribute to multiple stages of the evidence ecosystem and the ACE tool may therefore also be useful across these stages (Fig. 2) and to a wide range of stakeholders (Table 5).

**Table 5:** How the ACE tool may be useful to different stakeholders

Stakeholders	How the ACE tool may be useful
Citizens and the public	Supports the use in consultations and decisions of a wider range of sources of citizen views and perspectives
Health workers	Supports the use in consultations and decisions of a wider range of sources of health worker views and perspectives
Programme managers	Contributes to improving the content of programme reports and to facilitating critical appraisal of these sources
Evidence synthesis authors	Facilitates appropriate inclusion of unconventional source materials in syntheses (for example, those addressing questions such as the acceptability and feasibility of interventions and programmes, their equity and human rights impacts, and factors affecting their implementation), including in assessments of how much confidence to place in this evidence
Decision-makers at global, national and sub-national levels	Provides a structured and transparent assessment of the limitations of information from unconventional source materials contributing to a decision-making process, facilitating better use of this information
Staff of multilateral and bilateral agencies, global NGOs	Helps those developing and applying knowledge translation tools for guideline/guidance adaptation and adoption to understand strengths and limitations of the unconventional sources that they are using
Implementers at national and sub-national levels	Facilitates appropriate use of information from unconventional source materials in implementation planning

### Using the ACE tool in evidence syntheses

There are growing efforts to include unconventional source materials in evidence syntheses, including reviews addressing questions such as the acceptability and feasibility of interventions and programmes, their equity and human rights impacts, and factors affecting their implementation. The syntheses conducted for a recent WHO guideline on digital interventions for health systems strengthening provide one example of this [33, 34, 36]. These syntheses drew on information from programme development and implementation descriptions, feasibility and usability evaluations, programmatic observations and news articles to describe the range of strategies used to implement interventions considered in the guideline and to identify factors affecting implementation. Where unconventional source materials are included in a synthesis, assessments of the limitations of these source materials using the ACE tool may contribute to an assessment of confidence in the evidence included. For example, these assessments could feed into the GRADE-CERQual approach for assessing how much confidence to place in findings from syntheses of primary qualitative studies [27, 35]. GRADE-CERQual assesses confidence in the evidence on the basis of four key components: the methodological limitations of included studies; coherence of the review finding; adequacy of the data contributing to a review finding; and relevance of the included studies to the review question. While the ACE tool is not intended as a stand-alone tool to assess how much confidence to place in information from source materials, it provides the assessments needed for the methodological limitations component of GRADE-CERQual. The ACE tool therefore facilitates incorporation of these sources into evidence syntheses, which in turn may be used to develop recommendations and populate evidence briefs for policymaking or similar decision support tools.

### Using the ACE tool to support evidence-to-decision processes

As noted above, decision-makers often request information on the content of an intervention, programme or policy; how it might be implemented; and the factors that might affect this implementation. Unconventional source materials may provide important information on these issues, amongst others. Where these sources are used directly in documents to support evidence-to-decision processes, such as an evidence brief for policymaking [37] or an evidence-to-decision framework [11, 12, 38], the tool provides users with a structured and transparent assessment of the limitations of these sources. This, in turn, may help to ensure that these unconventional sources inform decision-making in an appropriate way. The ACE tool may therefore support efforts to widen the range of issues considered when developing clinical and health systems guidelines [38–42] and help to ensure that implementers’ perspectives are considered.

Organizations such as the WHO have started using evidence-to-decision frameworks such as INTEGRATE [38] that include components that may require consideration of diverse evidence sources. The recent WHO guideline on digital interventions for health systems strengthening [36] provides an example of the inclusion of unconventional source materials in evidence syntheses and of the use of an (earlier version) of the ACE tool to assess the methodological limitations of these sources [33, 34]. The two mixed method reviews included in this guideline show how these ACE tool assessments can feed into GRADE-CERQual assessments of confidence in the evidence and, from there, into Summary of Qualitative Findings Tables for use in an evidence-to-decision framework to support a guideline process [43].

Following guideline development, unconventional source materials can inform knowledge translation tools intended to facilitate the adaptation and adoption of guideline recommendations at national and subnational levels [14]. In this use context, unconventional sources may provide important information on contextual factors that affect the implementation of an intervention or programme, as well as on strategies that have been used in different settings to facilitate implementation and address potential barriers [44]. Here again, application of the ACE tool can help those developing and applying knowledge translation tools to understand strengths and limitations of the unconventional sources that they are using.

Beyond the guideline context, we anticipate that the ACE tool may help decision-makers and those who support them to make better use of local and other information from unconventional sources in their decision-making and in implementation planning [8]. Local evidence from unconventional source materials may be used in many ways to inform decisions, for instance, to describe local governance or financial arrangements for healthcare; to understand the size of a problem or health issue; to develop an implementation strategy; or to understand the possible equity impacts of a policy or programme following its implementation [8]. Information from unconventional source materials may also be helpful in planning the scaling up of interventions within a country or region [45]. In using this information, it is important to assess its strengths and limitations. In earlier work, we outlined a set of questions to guide assessment of the quality of local evidence [8]. The ACE tool builds on and extends these questions, focussing specifically on unconventional source materials. A number of initiatives across sectors have identified the need to make better use of local evidence (for example, [46–49]), and we believe that the ACE tool will contribute to improving both the quality and use in ‘routine’ decision-making of information on local conditions.

### Making better use of information from programme reports through applying the ACE tool

Programme reports are an important source of information on the design, implementation and monitoring of programmes, as well as on contextual factors affecting programme implementation. However, many programme reports do not provide sufficient detail on implementation and contextual factors, or provide this information in a very unstructured way. The WHO Programme Reporting Standards (PRS) checklist for sexual, reproductive, maternal, newborn, child and adolescent health (SRMNCAH) programmes was developed to improve programme reporting [17, 50] (see https://www.who.int/publications/i/item/WHO-MCA-17.11), and outlines key reporting items related to the design, context, development, implementation, and monitoring and evaluation processes of these programmes. It can be used across the life cycle of a programme, as it covers not only the reporting of processes and outcomes but also programme design and development. The PRS has been adapted by different users to meet the needs of specific types of programmes including social accountability [51]; for reporting on quality of care improvement processes [52]; and to guide the writing of country case studies on ‘Making multisectoral collaboration work’ [53].

The items included in the PRS informed the development of the ACE tool assessment criteria, particularly those relevant to the critical appraisal of information about programmes. The ACE tool, however, is intended to be applicable to assessing programme descriptions as well a wide range of other materials (Table 2). For many source materials, no reporting standards such as the PRS exist at this time. However, where SRMNCAH programmes have been reported using the PRS, this may facilitate critical appraisal of these sources using the ACE tool. We anticipate a virtuous cycle in which both the PRS checklist and the ACE tool may contribute to standardizing and improving programme reporting. This, in turn, should increase the usefulness of these reports for planning and decision-making as well for evidence syntheses. As noted above, the wider use in decision-making of the information included in programme reports has the potential to better address decision-makers’ questions regarding the feasibility of programmes, their equity and human rights impacts, and factors influencing their implementation. Better access to this wider range of information may help to ensure that decisions regarding health and other programmes are better informed and more appropriate.

### Facilitating the incorporation of a broader range of stakeholder perspectives in decision-making

Ensuring the inclusion of a broad range of stakeholder perspectives in decision processes at all levels is a moral imperative, and is also a key target within SDG 16 [54]. As noted earlier, unconventional sources can provide information on the views and experiences of people implementing, or affected by, a programme or intervention. This cannot replace the direct participation of stakeholders in decision-making, but can complement this participation and can provide critical information to those planning the intervention implementation. Recent debates on epistemic injustice in the ways in which knowledge is produced and used in health have highlighted the ways in which some groups of producers and users of knowledge are systematically marginalized [55]. For instance, the views and experiences of marginalized groups may sometimes be captured only in unconventional sources, such as blogs. These views may then be sidelined in contexts where other types and sources of knowledge are privileged and seen as more credible. In addition, researchers from or working with marginalized communities may experience systematic challenges in publishing their findings in conventional sources such as academic journals [56]. By contributing to the wider use in decision-making of unconventional sources, the ACE tool may help in small ways to rectify imbalances in broadening access to different perspectives and to promoting equity.

### Limitations of the ACE tool

The ACE tool has been developed and piloted using a relatively small number of source materials within the health sector. Wider application of the tool, including to source materials from sectors other than health, may identify additional signalling questions for each criteria or ways in which the clarity of the criteria can be improved. In addition, application of the tool is dependent on adequate description within source materials. Although we know little about how well unconventional source materials describe their subject, there is evidence of important gaps in descriptions within programme reports in the SRMNCAH field [50] and poor reporting is acknowledged widely in relation to intervention descriptions and other key information regarding the implementation of health interventions [16, 57, 58]. The range and nature of unconventional source materials (Table 2), and the lack of reporting standards for many of these, probably makes it more likely that these will not always include the information needed for assessment with the ACE tool. We hope, however, that wider use of the tool, and consequent greater use of these source materials in decision-making, will promote more comprehensive reporting over time.

As noted earlier, we developed the ACE tool using an approach used to develop other assessment tools [27, 28] and by drawing on the approach recommended for reporting guidelines by the EQUATOR Network [29]. However, we did not fully apply all of the recommended consensus methods for developing reporting guidance as some, such as multiple rounds of Delphi consensus processes, required resources beyond those available for this initiative. We think that the approach we used was able to incorporate feedback and advice from a wide range of potential users and experts, but it is possible that a formal Delphi process would have been helpful.

### Next steps in the application and evolution of the ACE tool

We envisage the following future steps to further understand ways of applying the ACE tool and to take its development forward:

- Wider application of the tool by a range of stakeholders (e.g. programme implementers, civil society) will help in understanding its usefulness to support decisions in a variety of contexts, including implementation and scale-up of interventions requiring various sources of contextualized knowledge. Wider application will also help to identify where the tool can be improved and may lead to further versions of the tool. We encourage users to share their feedback with us, and we particularly encourage colleagues from outside of the health sector to apply the tool as our experience to date is based largely on source materials related to health
- There is a need to document the application of the ACE tool to support decision-making in settings where conventional sources of evidence are not available or very limited
- We would like to document how the ACE tool can facilitate stakeholder engagement – including of health service users and civil society actors – and support the integration of community and family voices, thus contributing to people-centred decision-making [59, 60]
- To support the application of the tool, we would like to develop an explanation and elaboration (E&E) document, providing practical examples and guidance for each component
- We would like to explore ways of depicting the results of ACE tool assessments visually as this may improve the usability of these assessments within decision-making processes
- Where unconventional source materials are used within evidence syntheses, we need further work to understand how ACE tool assessments can feed into a broader assessment of confidence in the evidence from the synthesis. We also need more worked examples of the use of the tool to support decision-making as well as research to understand whether use of the tool facilitates more informed use of unconventional source materials
- We anticipate that it may be helpful to develop training materials for those planning to apply the ACE tool, and intend to explore this with potential users.

## Conclusions

Decision-makers have a wide array of questions and information needs when making decisions in relation to health and other interventions and programmes, and are keen to draw on information from their setting, and documented experiences from similar settings, in these decision processes. The ACE tool fills an important gap in the palette of tools for assessing the strengths and limitations of different kinds of policy-relevant evidence by providing explicit criteria for making these assessments in relation to unconventional source materials. We see the tool as important in helping us to use these sources in more appropriate and informed ways. Through doing so, the tool will facilitate efforts to strengthen and embed evidence-informed decision-making, including for complex policy and systems questions, and may be valuable within a wide range of decision processes, including (but not limited to) guideline development and implementation. We also hope that the tool will draw attention to the value of unconventional sources for decision-making and related activities, including helping to ensure that these are more responsive to the views and experiences of stakeholders, including those who are not well represented in other evidence sources. In the medium to long term, the ACE tool has the potential to also improve reporting within unconventional source materials, thereby improving their usefulness for decision-making and practice.

We encourage users to publish worked examples of both the application of the ACE tool to different types of unconventional source materials across different sectors and of ways in which these materials and ACE tool assessments have fed into decision-making processes. We welcome feedback that may help us improve the tool and the guidance for using it.

## Data Availability

This tool and the accompanying guidance are available here: https://zenodo.org/doi/10.5281/zenodo.7650035. Earlier versions of this tool were called the WEIRD (Ways of Evaluating Important and Relevant Data) tool and the END (Evaluating Non-conventional eviDence) tool. No other datasets were generated or analysed during the current study.
